# Application of Composite Bars in Wooden, Full-Scale, Innovative Engineering Products—Experimental and Numerical Study

**DOI:** 10.3390/ma17030730

**Published:** 2024-02-03

**Authors:** Agnieszka Wdowiak-Postulak, Grzegorz Świt, Ilona Dziedzic-Jagocka

**Affiliations:** 1 Department of Strength of Materials and Building Structures, Faculty of Civil Engineering and Architecture, Kielce University of Technology, al. Tysiąclecia Państwa Polskiego 7, 25-314 Kielce, Poland; gswit@tu.kielce.pl; 2 Department of Quality Management and Intellectual Property, Faculty of Management and Computer Modeling, Kielce University of Technology, al. Tysiąclecia Państwa Polskiego 7, 25-314 Kielce, Poland; i.dziedzic@tu.kielce.pl

**Keywords:** reinforcement, timber structures, bars, BFRP, GFRP, hybrid bars, flexural behavior, fire, environmental conditions, biological degradation, increased temperature, prestressing, FEM

## Abstract

The commercialization of modular timber products as cost-effective and lightweight components has resulted in innovative engineering products, e.g., glued laminated timber, laminated veneer lumber, I-beams, cross-laminated timber and solid timber joined with wedge joints. With the passage of time, timber structures can deteriorate, or new structural elements are required to increase the stiffness or load-bearing capacity in newly built structures, e.g., lintels over large-scale glazing or garages, or to reduce cross-sectional dimensions or save costly timber material while still achieving low weight. It is in such cases that repair or correct reinforcement is required. In this experimental and numerical study, the static performance of flexural timber beams reinforced with prestressed basalt BFRP, glass GFRP and hybrid glass–basalt fiber bars is shown. The experimental tests resulted in an increase in the load-carrying capacity of BFRP (44%), GFRP (33%) and hybrid bars (43%) and an increase in the stiffness of BFRP (28%), GFRP (24%) and hybrid bars (25%). In addition to this, glued laminated timber beams reinforced with prestressed basalt rods subjected to biological degradation, 7 years of weathering and prolonged exposure to various environmental conditions were examined, and an increase in the load-bearing capacity of 27% and an increase in stiffness of 28% were obtained. In addition, full-size laminated timber beams reinforced with prestressed basalt bars were investigated in the field as an exploratory test under fire conditions at elevated temperatures, and the effect of the physical–mechanical properties during the fire was examined via an analysis of these properties after the fire. In addition, a satisfactory correlation of the numerical simulations with the experimental studies was obtained. The differences were between 1.1% and 5.5%. The concordance was due to the fact that, in this study, the Young, Poisson and shear moduli were determined for all quality classes of sawn timber. Only a significant difference resulted in the numerical analysis for the beams exposed to fire under fire conditions. The experimental, theoretical and numerical analyses in this research were exploratory and will be expanded as directions for future research.

## 1. Introduction

The use of wood is currently increasing significantly, and it is a popular building material due to the fact that it has a wide range of lightweight construction applications. It is also characterized by its ease of production, its very good physical and mechanical properties and its low density. Environmental protection and low energy requirements are also among its advantages. This is why wood is increasingly being used as a construction material. Taking into account the orthotropic nature of wood, it is a complex material. The presence of knots, cracks or grain inclination, especially in the tension zone, has a significant influence on the behavior of a wooden member. Also, the properties of wood vary within the same species, and a description of many parameters is necessary to fully describe models of the behavior of wood.

It should be noted that fiber-reinforced polymer (FRP) materials are synthetic composites made from either high-strength fibers based on chemical synthesis (typically glass, carbon, aramid and basalt) or natural fiber-reinforced polymer (NFRP) composites, as well as hybrid fibers (a blend of synthetic and natural fibers) embedded in an adhesive matrix, e.g., epoxy, polyester and polyolefin.

The reinforcement elements can be available in various shapes and sizes. They are characterized by a very high strength and rigidity. It is worth considering that the repair of damaged timber is usually more cost-effective or less time-consuming, and it is FRP composite materials that have a high modulus of elasticity, strength or corrosion resistance [1,2,3,4].

It should be noted that composite materials have consistently played a significant role in construction, from the early civilizations to the present day. Above all, the main task has been to increase the strength, corrosion resistance and durability and to obtain a better strength-to-weight ratio [5,6,7,8,9]. Therefore, important and significant improvements in mechanical or thermal properties can be achieved by integrating reinforcements characterized by a high modulus and tensile strength into polymers [9,10]. FRPs are composed primarily of glass (GFRP), basalt (BFRP), carbon (CFRP) and aramid fibers incorporated into polymer matrix resins such as epoxy, polyester and vinyl ester resins. Typically, GFRP is mainly used because of its ability to achieve effective tensile strength compared to steel and its better cost-effectiveness than CFRP and BFRP. Unfortunately, a limitation of GFRP is its elastic modulus, which is significantly lower than that of structural steel, leading to an increased deformation of GFRP-reinforced components [11,12]. Much research has also been carried out using CFRP, BFRP, GFRP or steel fibers in various forms, including in tapes, mats and rods or in various other forms and characteristics of FRP, such as in construction, structures, reinforcing solid wood beams, wedge-jointed solid beams, laminated veneer lumber (LVL), cross-laminated timber (CLT) and glued laminated timber (GLT) beams—see [13,14,15,16,17,18,19,20].

Modern solutions also include engineered products such as glulam, formed from layers of graded structural lumber [13,14,15,16,17,18,19,20]. The advantages of these products include the use of shorter pieces of timber to form full-length laths using structural adhesives. They can then produce structural elements with different shapes and sizes with greater strength than their individual layers. Unfortunately, over time, timber structures deteriorate due to factors such as increased service loads, aging or biological degradation [21]. In this case, the use of repair or reinforcement instead of replacement is more beneficial. Traditional reinforcement techniques include the use of steel plates, aluminum plates or timber battens, but these may incur additional dead loads or reinforcement costs. In addition, steel components corrode under thermal stress. For this reason, FRP composites are an excellent solution for reinforcing timber structures, primarily due to their lightweight properties, while they can also achieve an increased stiffness or load-bearing capacity. Glued laminated timber beams are therefore excellent structural elements that are used in sports halls, swimming pools and public buildings. In modular timber frame construction, I-beams with laminated veneer lumber flanges are ideal because of their lightness, low cost and considerable load-bearing capacity or stiffness or due to the possibility of routing installation cables. On the other hand, beams made entirely of glued veneer, due to their higher cost, are only used as lintels for large areas of glazed windows, in openings above garages or as columns or foundations. But you cannot see them because they are placed inside, whereas glued laminated beams are visible. The GIROD (European glued-in rods) project involved several European countries with the aim of developing an acceptable standard, including fatigue testing, for connections based on glued-in rods in the context of Eurocode 5 [22]. The use of such connections has many advantages in obtaining high stiffness under axial loads, as well as excellent fire resistance, as a wood substance protects the bar and provides a good aesthetic appearance. Nowadays, glued-in rods are usually used, and several configurations of these connections can be found. They can also be used to repair rotten beam ends in traditional buildings [19]. In a research paper by Bengtsson and Johansson [23], structural applications of steel glued-in bars can be found. On the other hand, studies of laminated veneer beams reinforced with FRP and steel under different forms were shown in [24,25,26,27,28]. For example, Harvey and Ansell [24] from the University of Bath found that glass-fiber-reinforced plastic (GFRP) bars could be used as an alternative to steel bars. This is because GFRP pultruded bars have better compatibility with resin and wood, higher resistance to wet or acidic environments and better performance due to better bonding and weight reduction. Based on research, it was determined that the most suitable resin for bonding rods in laminated veneered wood (LVL) was an epoxy resin applied with a minimum glue joint thickness of 2 mm. It was also recommended that the surfaces of GFRP pultruded rods should first be treated by sandblasting or solvent wiping [24,25]. In addition, it was recommended that the minimum thickness of the adhesive bond should be 2 mm, to obtain the optimum static tensile strength of GFRP pultrusion in LVL. However, above this thickness, the strength does not change much. Broughton and Hutchinson [26] found that thicker bond lines between LVL steel bars increased the strength up to a thickness of 6 mm. In [27], the bond quality and joint performance between laminated veneer lumber (LVL) and metal panels were determined. Excellent results were obtained for specimens bonded to galvanized sheets and ChT and R plates. It should be noted that timber structures subjected to cyclic loading are now more commonly used in timber bridges, high-rise buildings or wind turbine towers. Unfortunately, however, no normative regulations are yet available for the fatigue of glued rods, resulting in incomplete or completely missing experimental data. Therefore, the author is currently researching glued laminated timber beams, solid beams, solid beams connected with wedge joints, cross-laminated timber beams, I-beams also with laminated veneer lumber, laminated veneer lumber beams reinforced with FRP materials, and steel in various forms and sizes and with different characteristics and structures, including in the form of rods made of various artificial fibers and natural fibers under cyclic load. In a study [28], it was found that low-cycle fatigue (LCF) resulted in wood and glue fracture, while rod failure was the limiting factor in high-cycle fatigue (HCF). Gentile et al. [29] investigated reinforced wooden beams with GFRP bars using the NSM technique. In their study, they obtained an increase in the flexural strength of the beam in the range of 18–46% with a percentage of reinforcement equal to 0.27–0.82% compared to unreinforced bars [29]. Raftery and Kelly [30] studied laminated timber beams reinforced with basalt FRP bars in the tension zone. Based on the results, the average increase in load-carrying capacity was 23%, and stiffness increased in the range of 8.4–10.3% [31]. Yang et al. [31] studied the reinforcement of glulam beams with steel, FRP bars and plates. They evaluated significant flexural strength and structural stiffness improvements, averaging up to 56.3 and 27.5%, respectively. In an experimental study [29], the flexural behavior of creosote-impregnated Douglas fir beams reinforced with glass-fiber-reinforced polymer (GFRP) rods was determined. Twenty-two half-scale and four full-scale GFRP-reinforced timber beams with reinforcement percentages ranging from 0.27 to 0.82% were tested for failure. The use of this reinforcement changed the mode of failure from brittle tensile failure to compressive failure, and the bending strength increased by 18 to 46%, eliminating the effect of local wood defects and increasing the bending strength of the elements. In a study [32], the impact of bonding different types of glass fiber reinforcement—in particular in the form of glass fiber fabric laminates—on the bending behavior of different compositions of timber elements—in particular sawn timber and glulam beams, consisting of spruce, pine and fir (SPF) No. 2, with dimensions of 140 mm × 140 mm × 2025 mm and 110 mm × 140 mm × 2025 mm, respectively—was determined. It was found that FRP fabric laminates increased the strength of lumber and glulam beams by up to 52% and 36%. Also, timber defects using FRP resulted in a change in the failure modes of unreinforced beams. In post-loading, the stresses were then transferred to the FRP bars. In subsequent research [33], it was determined whether using composites in the form of carbon fabric reinforcement or laminate strips for wooden beams would increase the load-bearing capacity of beams in bridges. Ten solid Douglas fir beams were taken from a timber stringer bridge in Yuma, Arizona, which was replaced in 1999. After testing, it was found that using carbon fabric on the timber beams provided a significant increase in flexural and shear strength and a nominal increase in beam stiffness. Another study [34] focused on four-point bending, where five beams were unreinforced and three were reinforced with two or four layers of straight and U-shaped FRP stressing threats. An additional load was created with a different maximum displacement with respect to the unreinforced beams. Results for simple pretensioned beams and reinforced U-shaped glulam beams showed increased strength and stiffness compared to unreinforced members [35]. However, the combination of span-to-depth ratio and insufficient development length resulted in shear failure. Inferior failure results were obtained by using transversely applied FRP sheets. Entirely confined beams were also the subject of this study in order to better understand the effect of the amount of reinforcement and to make a comparison with unreinforced beams. It was shown that the material model used to generate resistance curves achieved a model-to-experimental strength ratio of 0.92. In another study [36], the fiber reinforcement (FRP) of glulam beams subjected to simulated explosion loads was experimentally investigated. The strength and maximum deflection increases were in the ranges of 1.35–1.66 and 1.3–1.62, respectively. Likewise, the addition of FRP contributed to an increase in the tensile strain at break by 1.17 compared to the unreinforced beams.

It should also be noted that in European countries, such as Poland, due to the regulations in force here regarding reaction to fire and the spread of fire in multi-family buildings and public buildings such as offices, wood has been eliminated in structural form. But as mentioned above, in countries that pay special attention to the so-called “green” construction, in recent times, we have noticed an increasing use of wood in projects. However, the European leaders in this respect are Germany and the Scandinavian countries; e.g., in Norway, in 2015, a 14-story apartment building with a wooden structure was built, and in 2019, the construction of the highest (84.5 m) Mjøstårnet hotel on Lake Mjøsa was completed. In Canada, an 18-story wooden structure building was built on the campus of the University of British Columbia (excluding the cores of staircases, elevator shafts and the lowest floor, mainly made of reinforced concrete) [37]. In Polish regulations related to fire safety, specified in regulations [37,38,39], the key terms are as follows: fire resistance (properly made wooden elements usually achieve), fire spread (requires special treatment or protection from wooden elements), flammability (a feature related to the reaction to fire, which wood, even fire-retardant, is not able to achieve). When it comes to the scope of fire resistance, wooden elements are among the most complex and difficult to verify. Certain types of structures, such as beams and columns, can be calculated using the provisions of the standard [37,40]. Surface elements, e.g., walls and ceilings, are more difficult because the number of available solutions, e.g., cladding, is so large. Their impact is so different that work is still ongoing to select the appropriate calculation method beyond that described in ref. [40]. However, solutions where wood is used as a structure for other elements, e.g., partition walls, skylights, doors, and windows, which have a significant impact on fire resistance, pose even greater problems [37]. Then, it is very important to perform full-scale research. Therefore, as already written in published works [16,41,42,43], the next stage of these experimental, theoretical and numerical studies will be the study of the fire resistance of small-sized wooden elements (solid wood, laminated timber, cross-laminated timber, glued laminated timber beams, solid timber joined with glued joints, I-beams, laminated veneer lumber) reinforced with steel and FRP materials in various forms and structures, including different artificial fibers, natural fibers, etc. (FRP composite materials will be the external reinforcement; additionally, they will also constitute the internal reinforcement of the cross-section, so the same chopped fibers will be used and placed inside the cross-section as well as outside the cross-section); fire resistance of full-size wooden elements, including in the form of beams (solid wood, laminated wood, cross-laminated timber, solid wood joined with glued joints, glued laminated timber beams, I-beams, laminated veneer lumber) reinforced with steel and FRP materials in various forms and structures, including different artificial fibers and natural fibers (FRP composite materials will be the external reinforcement; additionally, they will also constitute the internal reinforcement of the cross-section, so the same chopped fibers will be used and placed inside the cross-section as well as outside the cross-section); and the impact of elevated temperatures on physical and mechanical properties—the behavior in the event of fire, as well as the physical and mechanical properties after fire of small- and full-size wooden elements will be examined, including in the form of beams (solid wood, laminated wood, glued laminated timber beams, cross-laminated timber, solid wood joined with glued joints, I-beams, laminated veneer lumber) reinforced with steel and FRP materials in various forms and structures, including different artificial fibers and natural fibers (FRP composite materials will be the external reinforcement; additionally, they will also constitute the internal reinforcement of the cross-section, so the same chopped fibers will be used and placed inside the cross-section as well as outside the cross-section). All these features will also be considered depending on the direction of fiber arrangement, structure of the wood element and reinforcement, as well as reinforced at individual technological stages of the production of wooden structural elements (solid wood, laminated wood, glued laminated timber beams, cross-laminated timber, solid wood joined with glued joints, I-beams, laminated veneer lumber) among others for fire protection and protection against long-term exposure to various environmental conditions. Research on the impact of biological degradation and 5-year exposure to atmospheric influences on reinforced wooden beams using composite and steel bars has been conducted for a long time and has long been demonstrated in published works [42,43]. In the works [42,43], apart from wooden beams, composite and steel bars were also subjected to long-term atmospheric influences in various weather and environmental conditions. In this study, the economic aspect was considered, and cheaper FRP materials such as basalt and glass were used. After innovative scientific, practical, economical and environmentally friendly was conducted research, satisfactory results were obtained. The full range of results of the used wooden beams subjected to 5 years of weathering and biological degradation as well as long-term operation in various environmental conditions and FRP materials subjected to long-term operation in different environmental conditions, their operation, and analysis are presented in the doctoral thesis [43].

These experimental, theoretical and numerical studies will be continued and will also be extended in the field of reinforced wooden beams for long-term operation in various environmental conditions of wood and reinforcement and their impact on the final result of combining reinforced structural beams (made of solid wood, glued laminated wood, glued laminated timber beams, cross-laminated timber, solid wood joined with glued joints, I-beams, laminated veneer lumber) as well as FRP reinforcements in various forms and structures as composite materials and chopped fibers, including different artificial fibers and natural fibers and steel (in various forms and structures and chopped fibers). These experimental, theoretical and numerical studies are also carried out under monotonic, static, dynamic, cyclic and repeatedly variable loads; long-term loads; environmentally variable loads; and exceptional loads on reinforced structural beams (made of solid wood, glued laminated wood, glued laminated timber beams, cross-laminated timber, solid wood joined with glued joints, I-beams, laminated veneer lumber) and FRP reinforcements in various forms and structures as composite materials and chopped fibers, including different artificial fibers and natural fibers and steel in various forms.

This paper investigates the behavior of reinforced bonded beams in the tension and compression zone under static loading with glued-in BFRP and GFRP bars. An identical degree of reinforcement for the bars used was assumed specifically for comparison purposes. Glass fibers have the advantage of being very well wettable by polymers, so the resin adheres easily to them. At the same time, the number of air voids at the interface is reduced to a minimum. This allows for high adhesion between the fibers and the surrounding polymer and an adhesive force between the two, ensuring a better bond. Glass fibers are characterized by their high tensile strength, lack of electrical conductivity and low thermal expansion. Their disadvantages include their tensile strength and modulus of elasticity decreasing at elevated temperatures. Glass fibers also creep under long-term loading. Their strength decreases over time, and they have less corrosion resistance than other fiber types. Basalt fibers are single-component materials obtained by melting solidified volcanic lava. It is important to consider that they are relatively inexpensive and have better physical and mechanical properties than glass fibers. Therefore, in addition to innovative and economical as well as practical and scientific research on glulam beams reinforced with prestressed composite bars as very cheap and easy-to-use materials in existing structures, the durability of wooden elements such as these bars was also shown. Moreover, due to the existing fire problem in wooden structures, especially multi-story ones, preliminary exploratory tests of reinforced beams in fire and post-fire conditions were presented. This is a very important study of the current legal and standard regulations. As mentioned above, the reinforcement application shown is practical, fast, easy and effective. All you need to do is make the grooves, prestress them, especially in the anchorage area, and then fill them with epoxy glue. Liquid epoxy glue is recommended to increase the adhesion of the ribbed bar and the wood, as it can penetrate into the wood’s pores.

Therefore, along with E-glass fibers, basalt fibers are the most widely used raw materials for composite bars. The main advantages of basalt fibers include fire resistance, the ability to damp internal vibrations, high sound insulation, high fatigue strength, a fiber service temperature of 982 °C, a fiber melting point of 1450 °C, high hardness and resistance to corrosion (including acid and alkaline corrosion).

Fibers also form the basis of the entire composite. Their characteristic properties include high strength and stiffness as well as low weight and size. This is due to the highly organized internal structure and the low probability of structural defects. The fibers used to construct FRP rods are glass, carbon, aramid and basalt fibers. Therefore, FRP rods with the appropriate type of fibers are used as reinforcement depending on the structural requirements (e.g., high strength, low deflection limit) or environmental requirements (e.g., chloride corrosion, high fire protection requirements). Fibers also differ in many properties, dependent on their production process or the types of particles they are made of. Aramid fibers are the lightest, carbon fibers are the most durable, glass fibers are the cheapest and basalt fibers are the most resistant to high temperatures. Also, all fibers are characterized by a typically linear stress–strain relationship. The modulus of elasticity depends not only on the diameter of a rod of one type (scale effect), the type of fibers used and the type of resin used, but also on the number and arrangement of fibers in the core, the physical and chemical match between the component materials of the rod and the production process and quality control carried out during production of composite bars. It should be noted that fibers and resin are quite resistant to creep in composite bars. For example, at elevated temperatures, reaching approximately 40% of the melting temperature of the polymer resin, thermosetting resins exhibit significant creep deformations under constant load. A very dangerous phenomenon is the sudden rupture of the composite reinforcement under the influence of creep deformations. This is related to the rod type; the direction, number and arrangement of fibers in the core; the fiber strength; and environmental conditions. However, the resin has much worse properties than the fibers. Conditions unfavorable for creep resistance include high temperature, exposure to ultraviolet rays, high alkalinity and cycles of moisture and drying, as well as freezing and melting. It was also found that rapid rupture will not occur when long-term stresses are limited to 60% of the early strength of FRP rod-reinforced elements. It should be noted that the phenomenon of rupture due to creep has not yet been fully investigated. There are no relevant conclusions yet regarding the behavior of structures reinforced with FRP bars for less than 100 h. Due to the insufficient understanding of the behavior of composite bars under long-term loading, it is often the high safety factors for creep that determine the size and amount of reinforcement needed (not the ultimate limit state). Therefore, further experimental, theoretical and numerical studies in this area will be continued, including testing of reinforced wooden beams, e.g., with composite and steel bars, under long-term loads, including the creep effect—the phenomenon of rupture. However, fatigue in composite materials is very complex because various fatigue effects may appear in the cross-section of the rod, such as resin cracks, fiber breaks, crack merging and component delamination. It should be noted that composite bars have relatively high fatigue strength. Moreover, the fibers themselves have virtually no fatigue resistance, but due to the good wettability of the polymers, the fiber–resin connection is strong enough to cope with load change cycles very well. However, the fatigue strength of composite bars is determined by the type of fibers and resin used, whether they are matched to each other, the adhesion between them, the number of fibers in the cross-section and environmental factors occurring during fatigue. It should be noted that the fatigue strength decreases with a decrease in the coefficient comparing the minimum stress with the maximum stress. Important issues are the limitation of cyclical displacements of the rod by the surrounding concrete or wood and the adhesion of the rod to concrete or wood. It should be noted that any embossments, recesses or other types of surface deformations improve adhesion but, at the same time, become places of stress concentration during fatigue loads. Also, fatigue is not yet fully understood because it depends on many factors in composite bars. It should be noted that the current design is based on components that rely on the production process, the environmental conditions in which the element will operate and the type of fatigue load to which it will be subjected. Therefore, experimental, theoretical and numerical research on wooden beams (made of solid wood, glued laminated wood, glued laminated timber beams, cross-laminated timber, solid wood joined with glued joints, I-beams, laminated veneer lumber) reinforced with FRP and steel and cyclical loads also continues. The issue of fatigue is also not fully understood yet because composite rods depend on many factors. Now, it is designed on components that rely on the production process, the environmental conditions in which the analyzed element will work and the type of fatigue load to which it will also be subjected. According to the ACI 440.1R-06 [44] standard, the thermal expansion coefficients of GFRP bars are as follows: linear: 6–10 × 10^−6^/°C; lateral: 21–23 × 10^−6^/°C. It should be noted that the standard does not provide thermal expansion coefficients for BFRP bars. It is assumed that they have similar properties, and we think expansion coefficients for BFRP rods are as follows: linear: 9–12 × 10^−6^/°C; transverse: 21–23 × 10^−6^/°C. The fire protection requirements for composite bars must be different in closed spaces, such as buildings, and in open spaces, such as bridges [45]. Likewise, it should be taken into account that using FRP bars to reinforce structures with high resistance to elevated temperatures is conditional on maintaining the structural integrity of the structure and is not advisable. The reason is the softening of the polymer material constituting the matrix and the outer cover of the rod under the influence of high temperature. This reduces the mechanical properties of the resin and increases its susceptibility to moisture absorption. It should be noted that irreversible changes in the internal structure due to particle movement occur after the glass transition temperature is exceeded. The value of the glass transition temperature depends on the type of resin. In composite materials, the fibers have much better resistance to high temperatures than the resin surrounding them. Therefore, they can carry significant longitudinal loads (tension). However, when the cooperation of individual fibers constituting the core of the bar is necessary—e.g., during bending and shearing—the load capacity reduction is much greater. Based on the research, it was found that due to the much greater resistance to high temperatures of fibers compared to resin, beams under long-term loads subjected to tests were destroyed when the composite reinforcement reached a temperature of 250–350 °C. It should be noted that the threshold temperature for fiber operation is approximately 880 °C for glass fibers and approximately 1250 °C for basalt fibers. Long-term exposure to negative temperatures may cause the matrix to harden, cause microcracks and microscratches and reduce the adhesion between the matrix and individual composite fibers. Likewise, freeze–thaw cycles combined with the action of salt may also result in the deterioration of the resin properties in the form of swelling of the polymer on the surface of the rod [45]. Likewise, changes over time in composite rods’ strength properties and stiffness depend on the environment in which they work. Different environmental conditions affect the bars before they are incorporated into the structure, during installation and after installation. It should be taken into account that the aggressiveness of the environment varies depending on the location of the element reinforced with FRP rods. Likewise, the properties of the bars may deteriorate, improve or remain unchanged. The environmental factors that affect the durability of FRP bars include water, heating and cooling cycles, freezing and thawing cycles, UV, higher temperature, acid solutions, alkali solutions, salt solutions and stresses in the rod. Therefore, composite bars’ durability is greater than steel’s, mainly due to the polymer resin constituting the surface layer of the bar, as it is in direct contact with the external environment. The composite rod must not degrade, and its three components must be sufficiently durable and interact appropriately with each other; these are the fibers, resin and cooperation surface between fibers and resin [45]. These are the factors that determine the susceptibility of a structure reinforced with FRP bars to degradation. These factors include covering of fibers by resin (wettability of fibers by resin), no scratches on the surface or in the cross-section of the rod, no air voids (smaller voids and larger spacing being better), internal solid bonds in the polymer structure of the resin (the production process must be properly controlled for the resin to achieve its strength properties) and strong adhesion between the fibers and the matrix (selecting the type of resin based on the type of rod so that the adhesion between the materials is as high as possible). Thus, the ends of the composite rod are a vulnerable place. If the bars are cut lengthwise, composite fibers will be visible in the final cross-section. Therefore, in this place, aggressive environmental factors can freely penetrate the rod along its length. It is equally important to properly seal the end sections of the FRP rods. Therefore, basalt rods are the most durable, while glass rods are the least durable. Therefore, the types of fibers differ in their resistance to environmental factors. At the same time, the disproportions in the longevity of composite rods are further deepened by the fact that polymer resins also react unequally to environmental aggressions depending on their composition. Moist and alkaline environments are unfavorable. However, this type of rod can also be made resistant by using appropriately shaped fibers, appropriate resin and an improved production process [45].

## 2. Materials and Methods

### 2.1. Materials

**Figure 1:**
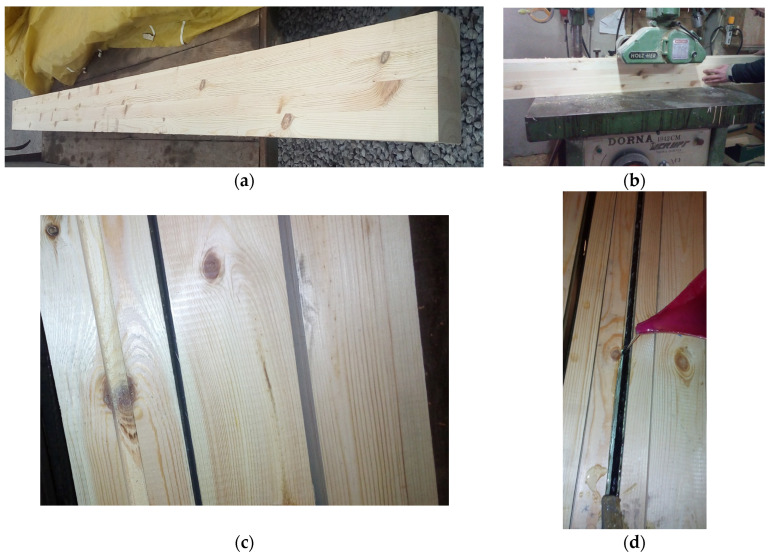
Production of 14 mm × 14 mm grooves for composite bar reinforcements: (**a**) glued laminated timber beam; (**b**) slot milling; (**c**) preparation of grooves to be filled with reinforcement; (**d**) filling prestressed basalt rods with glue.

The following materials were used in the experimental and numerical work:

Glued laminated timber: Twenty-eight beams made of glued laminated wood coming from the Małopolska Region Nature and Forest of Poland and from the beginning and end of the growing season were used, with final dimensions 82 mm × 162 mm × 3650 mm according to PN-EN 14080:2013-07 [46] class GL24c (external laths class T14, internal laths class T9), bending strength 24 MPa, shear strength 3.5 MPa, modulus of elasticity 11,000 MPa, shear modulus 650 MPa. The laths for testing were visually sorted, and all structural and geometric characteristics of the structural lumber were determined; KS—medium quality class of structural lumber; KG—lower quality class of structural lumber, Figure 1 [47], pine, spruce and fir species from Scandinavian countries. In addition, glued laminated timber beams subjected to biological degradation, 7 years of atmospheric influences and long-term exposure to various environmental conditions were used.

Reinforcing bars: BFRP basalt bars, GFRP glass bars, and glass–basalt hybrid bars were 10 mm in diameter and were introduced as reinforcement in the tension and compression zones for glued laminated beams. BFRP—experimentally determined elastic modulus 81.2 GPa, failure force 65,244 N, tensile strength 1521.3 MPa; GFRP—experimentally determined elastic modulus 62.3 GPa, failure force 51,312 N, tensile strength 1311.9 MPa; GFRP and BFRP hybrid bar—experimentally determined elastic modulus 77.1 GPa, failure force 52,675 N, tensile strength 1434.6 MPa. In addition, prestressed bars subjected to biological degradation, 7-year atmospheric influences and long-term exposure to various environmental conditions were used.

The epoxy resin for reinforced rods in the groove of glued beams was MapeWrap 31 [48]. It is a resin used to impregnate MapeWrap mats when reinforcing or repairing concrete structures, reinforced concrete or masonry elements using the dry method. MapeWrap 31 is a solvent-free epoxy resin with a gel consistency. Its parameters are as follows [48]: tensile strength ≥40 N/mm^2^, compressive strength ≥70 N/mm^2^, flexural strength ≥70 N/mm^2^, tensile modulus ≥2600 N/mm^2^. In addition, after gluing, the epoxy resin was subjected to biological degradation, seven years of atmospheric influences, and long-term exposure to various environmental conditions.

Figure 1 shows the preparation of glued laminated timber beams. The moisture content of artificially dried sawn timber was tested in industrial conditions at a sawmill. The humidity was 11.78%. The moisture content of the assortment was measured using a moisture meter type HT 65, manufactured by GANN (Gerlingen, Germany). The measurement was taken in the middle of the board width, at a distance of not less than 0.5 m from the face. The measurement sites were selected randomly. Measurements were not performed in places where defects and contamination occurred. The number of measurement places was three on each side of the sawn timber; in each place, the number of measurements was not less than three. The distance between individual measurement places was 10–15 mm. The measurement result was taken as the arithmetic mean of three measurements with the most similar values. 

Then, the structural timber was sorted visually according to the PN-D-94021:2013-10 standard [47]. The sawn timber’s thickness, width and length were measured during sorting. During the inspection of each piece of sawn timber, all structural and geometric features present in a given piece of sawn timber were measured, such as knots, fiber twist, cracks, resin blisters, plugs and fasteners, rot, insect spots, blue stain, hardwood, graininess, density, veneer, longitudinal curvatures of planes and sides, twist about width, transverse curvatures in relation to width, scratches, waviness of the cut, non-parallelism of planes and sides and non-perpendicularity of ends. In general, the most common indicator determining the quality class of sawn timber was knots, primarily their size and location on the cross-section of the sorted piece of sawn timber. The classes obtained were KS, a medium quality class, and KG, a lower quality class.

Later, the slats were glued. Each beam contained four layers, and the thickness of each layer after planing was approximately 40 mm, giving a total beam height of approximately 162 mm. Each lamella was cut from 4000 mm long timber. The slats were glued using D4 polyvinyl acetate glue. The gluing time was less than two hours. The slats were arranged so that the core was directed towards the upper part of the beam, according to PN-EN 14080:2013-07 [46].

The placing of the reinforcement was preceded by the milling of square grooves. The groove depth was 14 mm. The reinforcement in all grooves had a cover of approximately 2 mm and was connected to the wood using epoxy glue. The composite materials were cleaned with “Acetone” solvent before the glue was applied. It should be noted that the composite bars were fixed and prestressed (5 mm thick sheets and nuts were attached to the supports), and then epoxy glue was applied, filling the square grooves along the entire beam length. A minimum of 7 days was waited before laboratory tests to obtain the full strength of the reinforcement used.

### 2.2. Experimental Study

**Figure 2:**
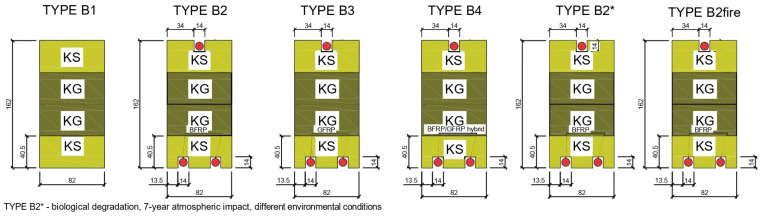
Reinforcement configurations of composite bars in glued laminated beams.

In the experimental part of the study, 28 full-size laminated beams in six groups were used for comparison with unreinforced beams. Five unreinforced beams were made as group B1; five reinforced beams prestressed with three basalt bars in the tension and compression zone with initial stress of 10 MPa were made as group B2; five reinforced beams prestressed with three glass rods in the tension and compression zone with a pretension of 10 MPa, as group B3; five reinforced beams prestressed with three glass–basalt rods in the tension and compression zone with a pretension of 10 MPa, as group B4; see Figure 2. In addition, five beams were made of glued laminated wood reinforced with prestressed basalt rods subjected to biological degradation, seven years of atmospheric influences and long-term operation in various environmental conditions, as B2* beams. Three glulam beams reinforced with prestressed bars were exposed to fire, and the reinforced beams were tested at elevated temperatures in fire and post-fire conditions to determine the physico-mechanical properties of their static work. The deformation of the bars was first examined before the bars were prestressed with a ‘Demec’ extensometer (Mayes $ Son, Vansittart Estate, Windsor, England, UK), and then, after the bars were prestressed, the deformation of the bars was again examined along the length of the bars with the extensometer, especially in the middle of the beam span, to analyze the elongation along the length of the beam. The groove was then filled with epoxy adhesive. Prestressing of the bars was carried out to give them straightness and better interaction with the wood and epoxy glue. Making the holes and filling them with bars alone without prestressing, filling them with bars with prestressing and epoxy glue, and fixing them at the ends of the beams on supports does not give an increase in reinforcement and reinforcement efficiency.

The experimental beams were tested in accordance with EN 408 + A1:2012 [49] using a bending test under static loading. Each beam was 3.65 m long with a rectangular cross-section of 82 mm × 162 mm. The percentage of reinforcement was 2.6 percent. The dimensions of the glued laminated beams were adapted to the static scheme according to EN 408 + A1:2012 [49]. The choice of this dimension was determined by the availability of timber in the local construction market. In these test models, it was important to use the top bars to reinforce the compressive stresses, for the reason that there is no significant difference between the compressive and tensile strengths.

**Figure 3:**
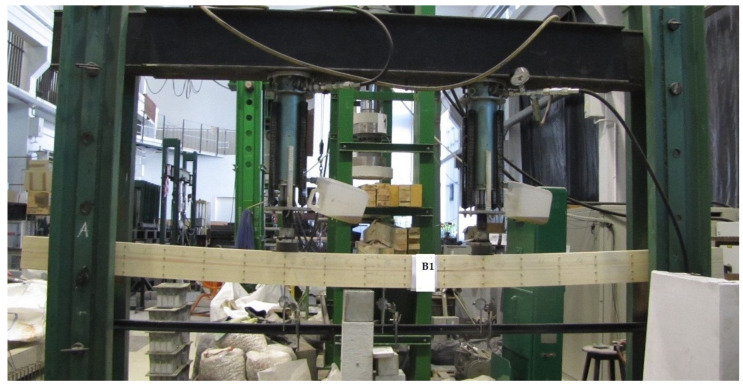
Schematic of the actual beams under test.

All prestressed full-size reinforced wooden beams with dimensions of 82 × 162 × 3650 mm^3^ were tested for four-point bending. To determine the bending mechanical properties in reinforced and unreinforced beams, the 4-point loading method was used. The tests were carried out on samples conditioned in air with a temperature of 20 ± 2 °C and relative humidity of 65 ± 5%. The load was applied at a constant feed rate and thrust to reach the maximum load after 300 ± 120 s. The loading force was tested; deformations over the entire surface of the beams over a length of 3000 mm between supports on two sides of the beams (which is very precise, considering there are usually tests in the literature only in the middle of the beam span) were tested using a Demec (Mayes $ Son, Vansittart Estate, Windsor, England, UK), 8-inch mechanical extensometer and 3 mechanical dial gauges over a length of 810 mm. All tests were carried out on a mechanically loaded testing machine. The test specimens were loaded to failure; all specimen elements were tested using a hydraulic machine with a capacity of 100 kN per actuator. During testing, the distance between supports was 3000 mm, and the distance between the loading forces and supports was 1000 mm. The experimental layout is shown in Figure 3.

**Figure 4:**
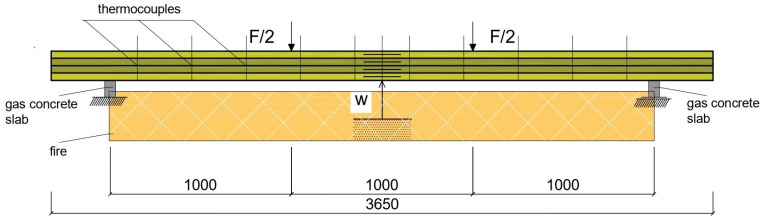
Test system for full-size reinforced beam type B2 at elevated fire temperatures.

In addition, the thermal properties of three reinforced B2 beams were examined at elevated temperatures as an exploratory determination of heat propagation and fire spread in reinforced wooden beams. The grooves and holes in the beams were filled only every 50 cm with epoxy glue and at the ends in the anchorage during prestressing. To ensure the safety of the experiment, experimental, theoretical and numerical tests were carried out in field conditions. Beams were placed on supports (aerated concrete bricks) and mechanically loaded, mechanical clock sensors were placed under the beam and the temperature inside the beams was measured. In field tests, the tested beams were surrounded by insulating material on the rear, left and right sides to reduce lateral heat flows during the heating process. Aerated concrete bricks were placed on the front surface of the beam, on which ten type K thermocouples were mounted at regular intervals of 30 cm. The temperatures ranged from 15 °C to 850 °C. Then, for safety reasons, the test prototype was turned off (Figure 4). Further research consisted of preliminary exploratory research aimed at determining the impact of increased temperature in fire conditions and physical and mechanical properties during and after the fire as a long-term action in various environmental conditions. Another aim was to determine whether the reinforcement protects the wooden element as internal reinforcement against fire. Therefore, the next stage will be research on small- and full-size elements with different arrangements of the wood structure as well as external or internal reinforcements such as FRP and steel, as well as chopped fiber. In the next laboratory stage, in addition to full-size wooden elements, it is planned to test small-size reinforced wooden elements in a chamber furnace with a very high operating temperature. After the reinforced samples are heated to specified temperatures, they will be tested statically and cyclically.

Additionally, for the B2 configuration, tests were carried out using wooden beams, subjected to biological degradation and seven years of weathering (e.g., water, snow, wind, temperature drop and rise), that were reinforced with basalt bars, which were also exposed to biological degradation and long-term operation in various environmental conditions. Therefore, the wood and the bars were subjected to biological degradation, seven years of atmospheric influences and long-term exposure to different environmental conditions. Environmental factors include water, ultraviolet radiation, elevated temperature, acid solutions, alkali solutions, salt solutions, heating and cooling cycles, freezing and thawing cycles and stresses in the rod.

### 2.3. Numerical Study

**Figure 5:**
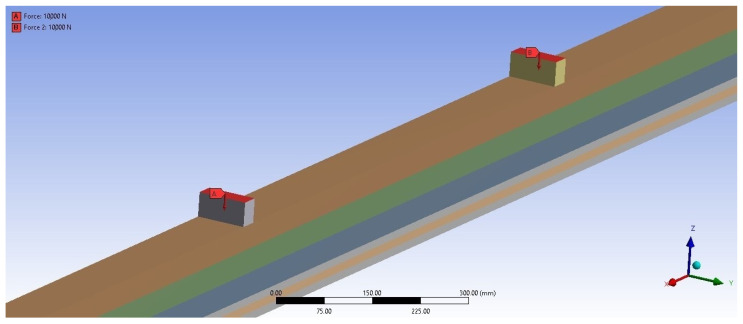
Finite element model—lamella geometry—various quality classes, glue between lamellas and rods, and rods.

Numerical modeling was performed with ANSYS 16.0, using the Static Structural module. Geometrical models of the beams were made using CATIA V5 and the finite element method. In the numerical studies, geometry was introduced for unreinforced as well as reinforced beams, so that the oriented geometry and reinforcement configurations identically reflect the beams tested experimentally. Also, boundary conditions were established using pinned or roller supports to eliminate vertical movement of the beams. The finite element mesh consisted of hexagonal and tetragonal elements. Hexagonal elements with a dimension equal to 10 mm were used on the lamella and support geometries. Tetragonal elements with a dimension equal to 5 mm were used on the rods and the glue area as hole filling and glue joints between the lamellas. The proposed finite element model is shown in Figure 5. The boundary conditions assumed in the numerical analysis were intended to reproduce a four-point beam’s bending faithfully. For this purpose, two concentrated forces of the same values were applied to the upper surfaces of the blocks. The supports were the lower blocks, placed at a greater distance between each other. One of the supports served as a fixed support, and the other served as a movable support. These assumptions were implemented using the Fixed Support function for the fixed support and Displacement for the movable one. Thermal analyses, including a temperature study, were designed for the reinforced beam. The numerical analysis also used the temperature in the experimental research to check the possibility of fire spreading over the entire surface of the beam and inside the beam. In the finite element method, the thermal analysis was performed in ANSYS in the Mechanical APDL module. Also, thermal visualizations were carried out and checked using the ‘Nodal temperature’ option. To determine the heat flow, the thermal properties of materials, namely the specific heat, thermal conductivity and density, must be given as a function of temperature, which is enabled by the Ansys program. The numerical model was also modeled in ANSYS. The finite element analysis involved heat flow in places exposed to fire and simulations in other places. This was a preliminary exploratory analysis as a direction for further research. Simulation fire action was modeled using preliminary experimental tests. To model beams loaded with fire, finite elements for thermal analysis were used as above. The dimensions of the finite element mesh were 10 mm and 5 mm. As mentioned, these were preliminary, exploratory studies and will be continued as directions for further research. The material data entered into the program are shown in Table 1 and Table 2.

## 3. Results

The results from experimental and numerical studies of the flexural properties of glued beams reinforced with prestressed FRP rods are shown. The experimental results of laminated beams are shown in terms of load-carrying capacity, failure mode, load–deflection and deformation.

### 3.1. Load–Deflection

**Figure 6:**
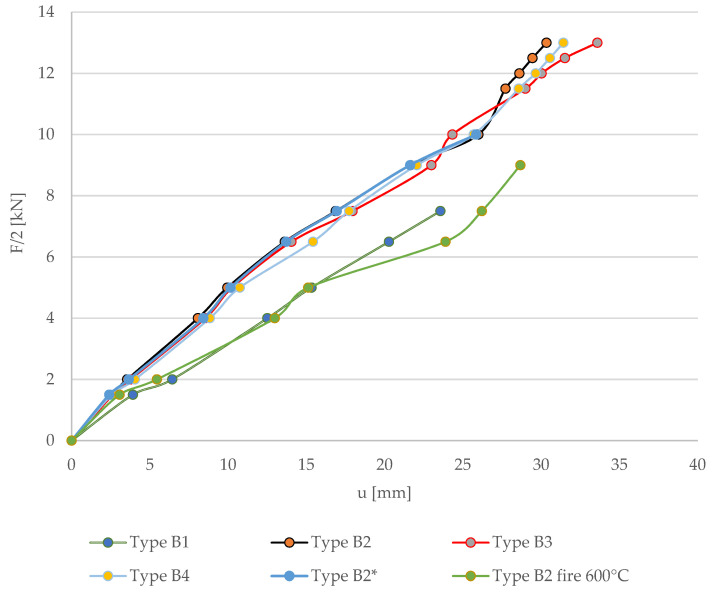
Load–displacement for all tested beams.

Based on the experimental bending tests, Figure 6 shows the load–deflection curves at the center of the beam span compared to unreinforced beams. From the tests, it was found that the use of reinforcing bars in the tension and compression zones of bonded beams improved the flexural strength and reduced the deflection values compared to the behavior of unreinforced beams in the range of 23.81–28.35%, improving the flexural stiffness. It is noted in the graph that the load–displacement curve for unreinforced B1 beams is linear, behaving in a virtually linear manner. It should also be noted that any deviation is due to the fact that the glued beams consist of different glued lamellas, which contain natural defects such as knots. However, in the case of biologically degraded wooden beams, subjected to seven years of atmospheric influences and long-term exposure to various environmental conditions (type B2*), the increase in stiffness was comparable to that for beams without degradation, without atmospheric influences and various environmental conditions (type B2), and amounted to 28.09% (F/2 = 7.5 kN). In the case of reinforced beams exposed to fire at initial loads (up to F/2 = 4 kN), the stiffness increased compared to unreinforced beams and amounted to approximately 21.68%. Then, at higher loads, deflections increased. For F/2 = 7.5 kN, the increase in beam stiffness at elevated temperature was 11.25% compared to unstrengthened beams without fire exposure. As mentioned, these were preliminary reconnaissance tests (field and outdoor tests); subsequent tests included laboratory tests of fire resistance, as well as the impact of elevated temperatures and checking the physico-mechanical properties after fire and in fire conditions.

### 3.2. The Maximum Bending Moment

In Table 3 below, the maximum bending moment results for beams unreinforced and reinforced with prestressed FRP basalt bars are shown.

The applied reinforcement in most glued laminated beams stiffened the beams, allowing them to carry higher loads. Therefore, the applied reinforcement leads to a combination of resulting horizontal shear and tensile failures, in contrast to unreinforced beams. The same satisfactory results were obtained for beams with biological degradation, atmospheric influences and long-term operation in various environmental conditions, as much as a 26.90 increase in load-bearing capacity. However, when it comes to field tests as reconnaissance tests on the effects of fire, the impact of increased temperatures on reinforced beam elements as well as the wood materials themselves and the reinforcement used, searching for appropriate protective and fire-retardant reinforcement, maintaining appropriate fire resistance, obtaining the assumed reaction to fire, examining the spread of fire and then conducting theoretical and numerical analyses, as well as analysis of physico-mechanical properties after fire and in fire conditions, they will be extended as directions for further research.

### 3.3. The Image of the Destruction of the Beams

**Figure 7:**
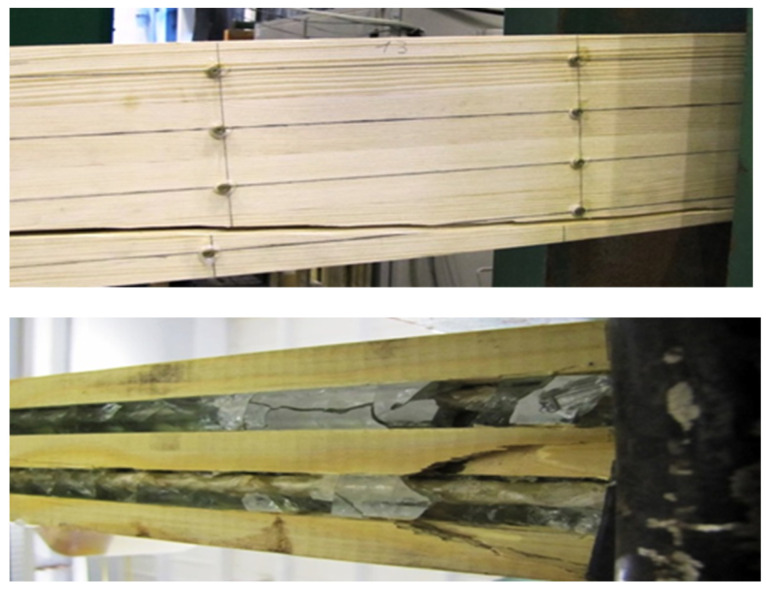
An image of the destruction of the reinforced beams—B3-4.

The failure pattern of the glued beams varied for each beam type. This was due to the loading system, the timber material’s heterogeneity, the structural and geometric features’ magnitude and knots. Therefore, the basic stages of failure included the following:

- Rapid failure in the tension zone in the unreinforced element due to the presence of knots; the tensile stress in the extreme tensile fibers exceeded the tensile strength limit of the timber.
- Usually, in elements reinforced with prestressed composite rods in the tension and compression zones, rapid and sudden shear failure occurred along the wood fibers after the glulam shear strength limit was exceeded; see Figure 7.

In most of the reinforced beams, there was shear damage, sometimes delamination, and cracks in the epoxy resin appeared. It was also noted that the reinforcement also prevented damage and inhibited crack propagation in the timber.

### 3.4. Deformation of Wood at the Height of the Beam Section

**Figure 8:**
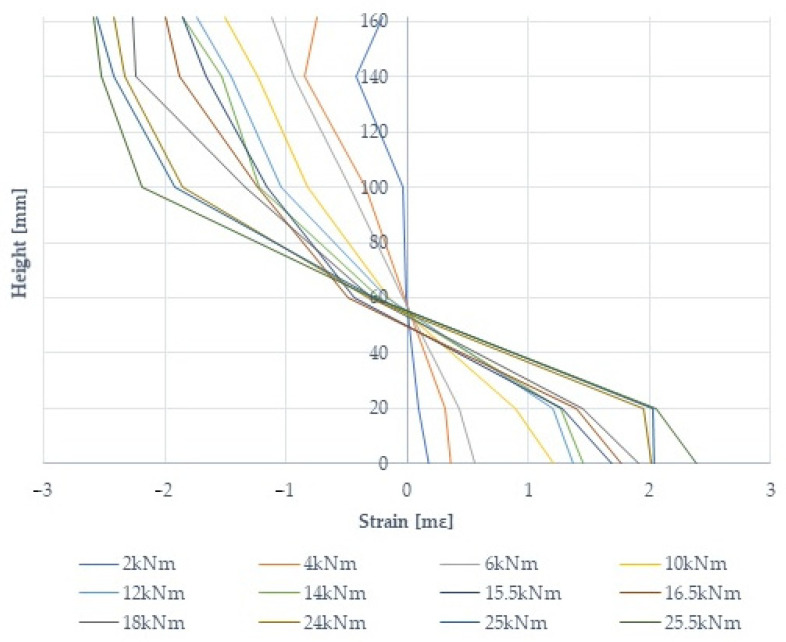
A diagram of timber deformation at the height of the timber section at the center of the B2-3 beam span.

Unreinforced type B1 beams showed tensile strains (with normal stresses) greater than those for unreinforced type B2, B3, B4, B2* and B2fire beams. Below is a graph of the wood deformation at the height of the beam section in a beam reinforced with prestressed basalt bars, B2-3; see Figure 8. The application of reinforcement significantly reduced the tensile stresses by up to 22% and the compressive stresses by 15%. In the areas of wood defects (knots, fiber twists, cracks, resin blisters, etc.), increases in the deformation and stresses of the wood and fiber composite were noted.

### 3.5. Experimental and Numerical Studies

**Figure 9:**
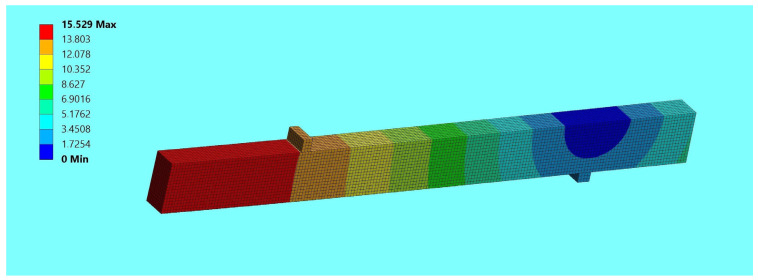
Displacement image using the finite element method for beam B1—Ansys.

**Figure 10:**
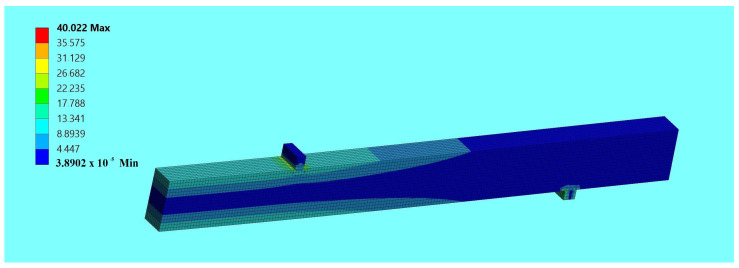
Normal stress image using the finite element method for beam B1—Ansys.

In the numerical study, a three-dimensional finite element model was defined to determine the behavior of unreinforced and reinforced beams, made with different configurations of wood quality classes, reinforced with composite bars. Numerical investigations included checking and comparing the results of unreinforced beam elements with reinforced elements in the laboratory and numerical analysis. The experimental and numerical results obtained were compared to verify the accuracy of the finite element models for a force F/2 equal to 5 kN; see Table 4 and Table 5. Figure 9 shows an example of a finite element numerical displacement analysis for beam B1, while the finite element normal stresses are shown in Figure 10.

Based on the results obtained, it was concluded that numerical analysis can be applied to reinforced elements to design different reinforcement schemes with particular attention to the configuration of wood quality classes. In the above study, a three-dimensional finite element model was defined that determined the stiffness of unreinforced and reinforced beams with reasonable accuracy. The model obtained was consistent with experimental studies; the stiffness of reinforced beams can be increased by using different configurations of lamella quality classes and composite bar placement. In the numerical studies, a satisfactory agreement was obtained in the materials tested, in the timber and the composite bar, and in particular, in the consistency of the results. This is because Young’s modulus, Poisson’s modulus and the shear modulus for all quality classes of structural lumber were determined experimentally for the numerical analysis. The only significant difference resulted in beams at increased fire temperature, but these numerical tests will also be further extended.

## 4. Discussion

Experimental and numerical studies show the static behavior of beams reinforced with various prestressed BFRP fibers. Additionally, the tests used beams and bars subjected to biological degradation, seven years of atmospheric influences and various long-term environmental conditions. The next part also presents experimental research in field conditions as an exploratory study of exposure to fire at elevated temperatures in fire and post-fire conditions. The force–deflection diagram shows that most of the beams show a linear relationship, which means that the deformations in prestressed bars increase linearly for the most part. Composite rods, however, do not have the ability, of course, after exceeding certain stresses, to strengthen plastically or to redistribute internal forces. It should be noted that this is different from steel. Based on the research, it can be concluded that the reinforcement with prestressed composite bars does not show any increase in plastic deformation, which would indicate an impending disaster. As you can see, the deformation increases until it exceeds a certain value, and then brittle failure of the structural element occurs. Therefore, in the process of designing structures, it also happens that it is not the criteria of the limit state of stresses and strains but the fatigue resistance and the resistance to sudden breakage of the bar—creep—that are determining factors [45].

It should also be remembered that using FRP bars to reinforce structures, where high resistance to elevated temperatures is a necessary condition for maintaining the structural integrity of the structure, is not recommended. Similarly, in the tests, because the fibers themselves in composite materials have much better resistance to high temperatures than the resin surrounding them, in the case of fire-reinforced beams, attempts were made to fill only the holes every 50 cm and at the anchor point with epoxy glue. In future research, it is also planned to investigate, in addition, only composite staple fibers used in the internal and external cross-section of the wooden element, in addition to other FRP materials and steel. Then, they can carry significant longitudinal loads (tension). In tests on beams made of wood laminated with prestressed basalt rods, the epoxy glue determined the adhesion of the rod to the wood. But when the basalt bar’s temperature limit was reached, the epoxy adhesive was no longer able to transfer the stresses from the wood to the bar’s core. It was noticeable that when the temperature was above 200 degrees Celsius, the adhesion decreased by up to 65%. Here, we can extend the tests under long-term load by acting on fiber-reinforced beams, and then we will obtain a higher temperature threshold and a decrease in adhesion, even assuming 400 degrees Celsius. These were reconnaissance tests, as mentioned, and a sudden increase in deflections occurred starting from F/2 = 4 kN. Then, there was a decrease in the adhesion of the bars to the wood, but the limit temperature of the glued wood was still reached at 600 degrees Celsius, for a time of approximately one hour. It was still possible to test the sample, but for safety reasons, everything was dismantled and secured; only then were the physical and mechanical properties after the fire additionally tested. These were exploratory studies; subsequent extended studies were designated as directions for further research. Also, during these experimental tests, it was observed that excessive beam deflections and significant cracks suddenly occurred due to the deterioration of the anchoring quality as the temperature increased. It was also noticed that the physical and mechanical properties of the composite rods deteriorated with increasing temperature, but if they did not exceed the glass transition temperature of the fibers, they returned almost completely to their initial values [45].

It should also be remembered that in prestressed structures, additional stresses occurred at the bar–wood interface in these tests due to the relatively high coefficient of transverse thermal expansion. Similarly, during a fire, the flame had access to the rod and caused the formation of toxic fumes when burning the polymer resin. But then gases were released, which insulated the rod and made it difficult for the flame to penetrate deeper into its structure. If a cover is made, there is no access to oxygen needed for oxidation, and therefore, the structure does not burn despite high temperatures. Similarly, it was noticed that long-term exposure to negative temperature may also cause hardening of the matrix, microscratches, microcracks or reduced adhesion between the matrix and individual FRP fibers. Freeze–thaw cycles during various long-term environmental conditions and the effect of salt also manifested themselves in the deterioration of the resin properties as the polymer swelled on the rod surface—but this was only observed in two rods.

Therefore, to prevent the reduction in adhesion between FRP rods and wood as a result of the expansion and contraction of the rods under the influence of temperature cycles and degradation, e.g., resulting from frost, the rod cover and composite building blocks should be selected appropriately depending on the type of rod and the element’s operating environment. In the case of securing the structure at elevated temperatures, it is necessary to select a cover that protects the rod against the influence of high temperatures. Likewise, the type of polymer resin material and the type and size of fibers in the rods must be sufficiently known because, of course, the critical temperatures of the materials—the glass transition temperature of the resin and the threshold temperature at which the fibers lose their strength properties— are decisive here, affecting the designed operating temperature of the structure and its fire resistance [45].

## 5. Conclusions

On the basis of the results of the experimental and numerical tests and analyses of static work efficiency, load capacity, stiffness, deformations at average and elevated temperatures and various environmental conditions carried out on beams glued in layers with prestressed composite bars, the following conclusions were drawn:

- The use of prestressed composite bars to reinforce timber beams is an effective way of increasing the load-bearing capacity and stiffness of members used in both existing and newly designed structures.
- The highest load-bearing capacity and stiffness increases were obtained for beams reinforced with prestressed basalt bars. The increase in load capacity was 44%, and that in stiffness was 28%. It should also be noted that comparable efficiencies were obtained for glued laminated beams reinforced with prestressed glass–basalt bars, so-called hybrid bars (increase in load capacity—43%; stiffness—25%). Therefore, it is possible to reduce the production costs of basalt bars by producing hybrid bars using winding, ribbed basalt fibers wound on glass fiber.
- A decrease in compressive and tensile strains was observed in all types of reinforced bonded beams. Reinforcement significantly reduced tensile stresses by 19–22% and compressive stresses by 11–15%. The presence of composite bars inhibits or limits crack propagation.
- For reinforced beam elements made of glued laminated timber of classes KS and KG, the most common form of failure was shearing of the timber along the fibers. For unreinforced beams, the failure usually occurred in the tension zone as a result of the fracturing of the timber fibers near timber defects or hidden defects—knots. Therefore, it was observed that prestressed composite bars improved the interaction between the cracking knot and the stiffening ‘glue-bar’ joint.
- In the above study, a three-dimensional finite element model was defined which determined the stiffness of unreinforced and reinforced beams with reasonable accuracy. The model obtained was consistent with the experimental studies. The differences were at the level of 1.1–5.5%. Among other things, satisfactory agreement was obtained by using experimental tests to determine the Young’s modulus, Poisson’s modulus and shear modulus for the KS and KG classes of structural lumber quality forming the laths of the laminated beams. A significant difference of as much as 11.8% concerned only beams at elevated temperatures in fire conditions. These were only exploratory tests. They will be extended as directions for further experimental, theoretical and numerical research on small-size and full-size wooden beams (including solid beams, solid ones joined with glued joints, glued laminated timber, laminated veneer lumber, I-beams (with laminated veneer lumber and also solid wood), glued laminated beams, cross-laminated timber) reinforced in various forms at every production and technological stage with different FRP fibers (including various artificial and natural fibers) and steel as external and internal reinforcement or as staple fibers, at elevated temperatures, in fire conditions, after fire, under the influence of fire, reacting to fire, in fire spread and standard fire resistance tests, in long-term operation in various environmental conditions (to study their load-bearing capacity, stiffness, deformability and acoustic emission) and as structural elements of various structures with various fiber shapes subjected to static, cyclic, long-term and variable loads—bending, shear, compression, tension.
- FRP-reinforced timber beams subjected to tension zone stress at elevated temperatures typically failed through anchorage degradation. This was due to the loss of their properties due to exposure to high temperatures or to the basalt polymer, e.g., softening, or due to the basalt fibers exceeding the threshold temperatures for fibers. Therefore, due to the deterioration of anchoring quality as the temperature increased, excessive beam deflections and crack widths that exceeded the norm possibly occurred.
- In prestressed structures, it should also be considered that the high coefficient of transverse thermal expansion causes additional stresses at the rod–adhesive and rod–wood interfaces. Then, during a fire, the flame that has access to the rod causes the formation of toxic fumes while burning the polymer resin. But it should also be noted that gases may then be released, insulating the rod and preventing the flame from penetrating deeper into its structure. But the bars in the structures are also located inside the wood—there is no access to oxygen needed for oxidation, and they do not burn despite the high temperature.
- It should be noted that despite biological degradation, atmospheric influences and long-term exposure to various environmental conditions, very satisfactory load-bearing capacity (27%) and stiffness (28%) results were obtained. Therefore, the durability of composite bars is greater than the durability of steel. This is because the polymer resin, as the surface layer of the rod, does not allow aggressive factors to penetrate into the composite. This research program was planned to be expanded as a direction for further research, taking into account various long-term atmospheric influences and environmental conditions of the beams and the reinforcement used.
- It should also be taken into account that in numerical modeling, we treat wood as an orthotropic material; in reality, it is a heterogeneous material—anisotropic. Therefore, any defects in the wood are very much ‘averaged out’. And yet, wood defects, especially knots and defects occurring in the tension zone, primarily determine the load-bearing capacity or stiffness of the flexural member.
- Thus, the applied reinforcement technique can be useful in innovative wood products and engineering for reinforcement system analysis and repair.
- Analytical methods for predicting the strength of reinforced beams are imprecise due to the very high heterogeneity of natural wood material, indicating the need to use more full-size technical-scale elements per trial to reduce the error tolerance. Also, due to the significant heterogeneity of wood materials, full-size elements should be examined on a technical scale; the scale effect in wood materials is unreliable.

## Figures and Tables

**Table 1:** The material data for the reinforcing elements.

Material	MOE (MPa)			Poisson’s Ratio			Shear Modulus (MPa)		
	L	R	T	L	R	T	LR	RT	LT
Epoxy resin	2600	78	78	0.3	0.015	0.3	-	-	-
GFRP	62,300	1880	1880	0.25	0.0125	0.25	-	-	-
BFRP/GFRP	77,100	2327	2327	0.22	0.011	0.22	-	-	-
BFRP	81,200	2451	2451	0.19	0.0095	0.19			

**Table 2:** The material data for the lamellas of the glued laminated beam.

Quality Class for Structural Sawn Timber	Young’s Modulus (MPa)			Poisson Modulus						Shear Modulus (MPa)		
	L	R	T	LR	LT	RT	TR	RL	TL	LR	LT	RT
KG	9130	3013	3013	0.45	0.49	0.56	0.35	0.044	0.018	550	550	55
KS	11700	386	386	0.48	0.51	0.60	0.37	0.048	0.019	880	880	88

**Table 3:** The bending moments (M_max_) for beam types “B1”, “B2”, “B3”, “B4”,”B2*”, “B2fire”.

BEAM B1	M_max_ [kNm]	BEAM B2	M_max_ [kNm]	BEAM B3	Mmax [kNm]	BEAM B4	Mmax [kNm]	BEAM B2*	Mmax [kNm]	BEAM B2fire	Mmax [kNm]
B1-1	18.50	B2-1	27.00	B3-1	22.50	B4-1	24.00	B2-1*	19.50	B2fire-1	18.00
B1-2	16.50	B2-2	22.00	B3-2	24.00	B4-2	22.50	B2-2*	24.00	B2fire-2	16.50
B1-3	17.00	B2-3	25.50	B3-3	21.00	B4-3	28.00	B2-3*	21.00	B2fire-3	17.00
B1-4	15.50	B2-4	26.00	B3-4	24.50	B4-4	24.50	B2-4*	23.50		
B1-5	18.00	B2-5	23.00	B3-5	22.00	B4-5	23.00	B2-5*	20.50		
Average	17.10	Average	24.70	Average	22.80	Average	24.40	Average	21.70	Average	17.17
Increase [%]	-	Increase [%]	44.44	Increase [%]	33.33	Increase [%]	42.69	Increase [%]	26.90	Increase [%]	0.39

**Table 4:** The comparison of laboratory and numerical results of the tested beams; displacements—F/2 = 5 kN.

Type	Experimental Study—Deflection [mm]	Numerical Study—Deflection [mm]	Difference [%]
B1	15.33	15.5	1.1
B2	9.95	10.5	5.5
B3	10.26	10.7	4.3
B4	10.75	11.3	5.1
B2*	10.15	10.7	5.4
B2fire	15.12	16.9	11.8

**Table 5:** The comparison of laboratory and numerical results of the tested beams; tensile stresses—F/2 = 5 kN.

Type	Experimental Study—Normal Stress [MPa]	Numerical Study—Normal Stress [MPa]	Difference [%]
B1	36.11	40.0	1.1
B2	31.15	37.4	2.0
B3	33.07	38.9	1.8
B4	32.51	37.8	1.6
B2*	33.12	-	-

## Data Availability

Data are contained within the article.
